# Supplementary material for: Mixed Milk Feeding: A New Approach to Describe Feeding Patterns in the First Year of Life Based on Individual Participant Data from Two Randomised Controlled Trials
Source: Nutrients. 2022 May 24;14(11):2190. doi: 10.3390/nu14112190 (PMC9182968; doi:10.3390/nu14112190)
Supplement: Supplementary file 1 [file nutrients-14-02190-s001.zip › nutrients-1679118-supplementary.pdf]

nutrients

MDPI

Supplementary Material

Supplementary material - Mixed Milk Feeding: A New Approach to Describe Feeding Patterns in the First Year of Life Based on Individual Participant Data from Two Randomised Controlled Trials

1. Tempo – Study Description

1.1. Methods

This prospective, randomized, controlled, multi-center study was conducted in 13 different countries in Europe and Asia (Belgium, Czech Republic, Germany, Hong Kong, Hungary, Israel, Italy, Singapore, Slovakia, Spain, Taiwan, the Netherlands, and United Kingdom). The study was registered in the clinicaltrials.gov registry with identifier: NCT03067714 on March 1st 2017.

1.2. Study population

Healthy term infants at high risk of developing allergy (based on family history of allergy), aged maximum 16 weeks of age, were included in the study from March 2017 till March 2019. Infants who consumed infant formula based on intact protein (with the exception of the first 72 hours after birth) and/or added probiotics or probiotic supplements, participating in another clinical study or whose parents were judged unable to comply with the protocol requirements were excluded. Infants who suffered from existing allergic manifestations, suspected cow's milk allergy, lactose intolerance, galactosaemia, severe congenital abnormality, severe neonatal illness, known underlying disease predisposing to infection, or severe renal and hepatic failure were also excluded.

1.3. Study design

In randomized infants formula intake was started before the age of 16 weeks (combination with breastfeeding allowed) and continued until the age of 12 months. Infants whose mother had the intention to exclusively breastfeed the infant for at least 16 weeks were eligible to participate in the breastfed reference group. Subjects were required to visit the site at 17, 26, 39, and 52 weeks of age for the assessment of allergic manifestations and collection of anthropometric and other relevant medical data. Parents were asked to maintain an (electronic) diary from enrolment onwards to record data on study product intake and breastfeeding, introduction of complementary feeding, and gastro-intestinal tolerance. Additionally, a skin prick test was performed and a blood sample was taken at the age of 52 weeks. Faecal samples were collected shortly after screening (breastfed reference group) or before starting study product intake (randomised groups), and at 17, 25, 39 and 52 weeks of age. Saliva samples as well as nasopharyngeal swabs were collected at screening, and at 26 and 52 weeks of age.

1.4. Formulae

Both test and control product were infant formulas for infants until the age of 12 months. The test product was a nutritionally complete partially hydrolysed whey protein based formula enriched with a mixture of prebiotic short-chain galacto-oligosaccharides (scGOS), and long-chain fructo-oligosaccharides (lcFOS) and Bifidobacterium breve M-16V. The control product was a nutritionally complete infant formula based on intact cow's milk proteins.

1.5. Ethics

The approval of the relevant ethics committees in the participating countries was obtained before the start of the study. The study was conducted according to ICH-GCP principles, and in compliance with the principles of the 'Declaration of Helsinki' (59th WMA General Assembly, Seoul, October 2008) and with the local laws and regulations of the country where the study was performed. Written informed consent was obtained from all parent(s)/guardian(s) before enrolment in the study.

2. Supplementary material – Figures

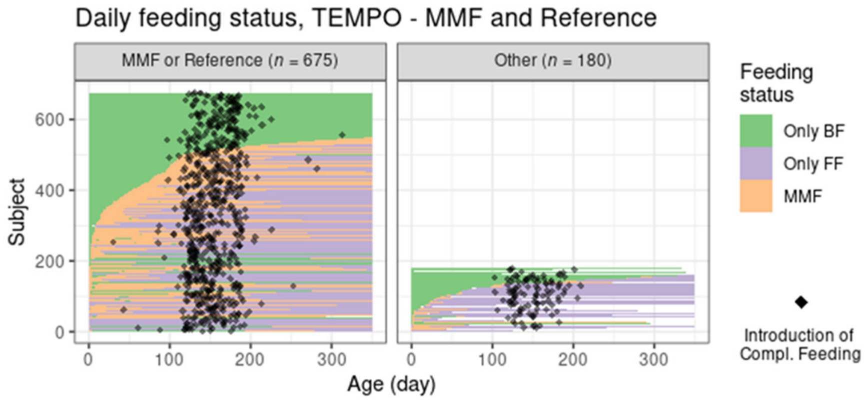

Figure S1. Feeding pattern of each subject in Tempo. Every subject's feeding pattern is represented by a horizontal line, the colour indicating the type of feeding at a given age. Left, the MMF and Reference groups. Right, the "Other" group, comprising subjects that either had very little feeding data or short transitions between exclusive breastfeeding and exclusive formula feeding.

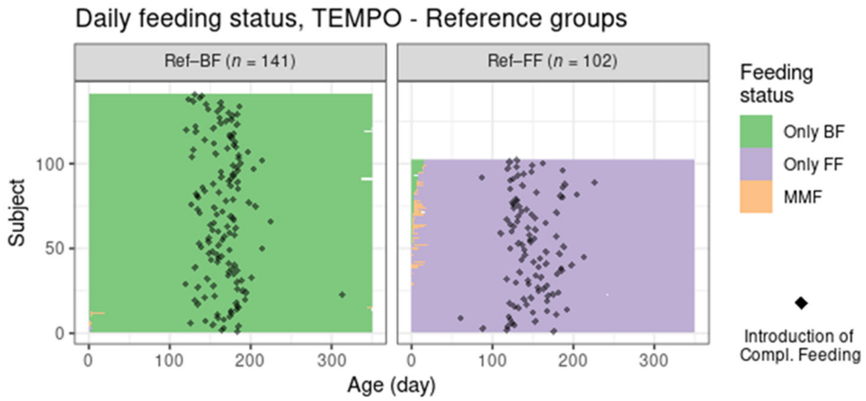

Figure S2. Feeding pattern of each subject in the reference groups in TEMPO. Every subject's feeding pattern is represented by a horizontal line, the colour indicating the type of feeding at a given age.

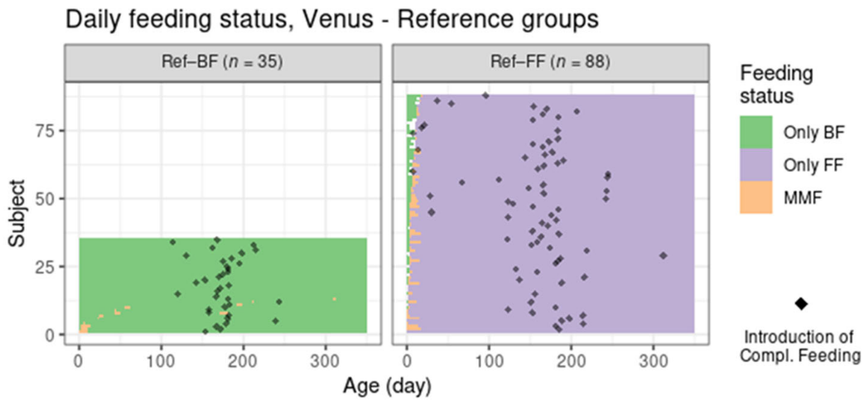

Figure S3. Feeding pattern of each subject in the reference groups in Venus. Every subject's feeding pattern is represented by a horizontal line, the colour indicating the type of feeding at a given age.

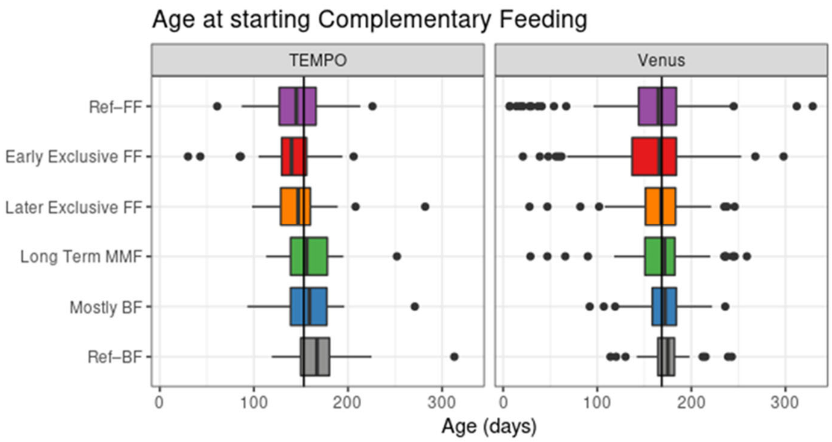

Figure S4. Age at starting Complementary feeding (boxplots), in TEMPO and Venus. The middle line shows the median, the limits of the box show the 25% and 75% quantiles of the distribution.

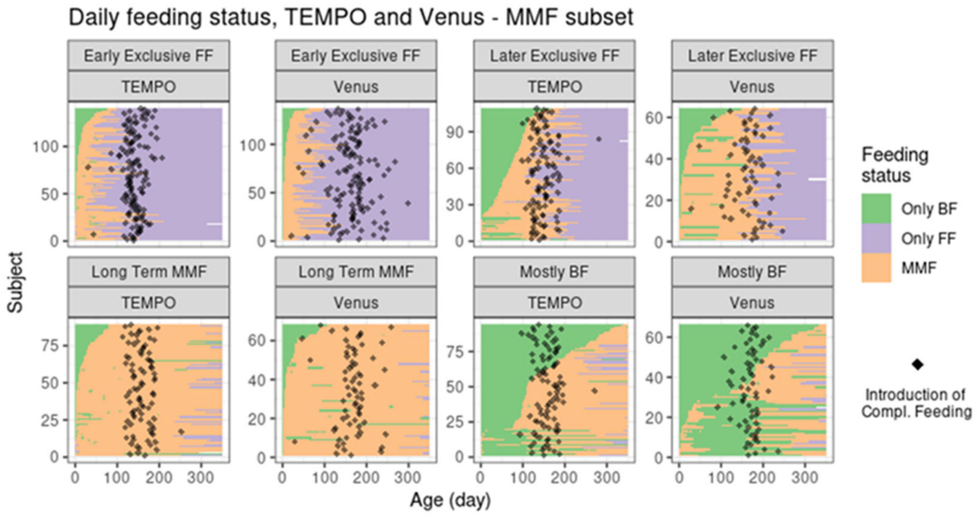

Figure S5. Feeding pattern of each subject in the MMF Clusters in TEMPO and Venus. Every subject's feeding pattern is represented by a horizontal line, the colour indicating the type of feeding at a given age. Note that the scales on the vertical axis are different for each box.

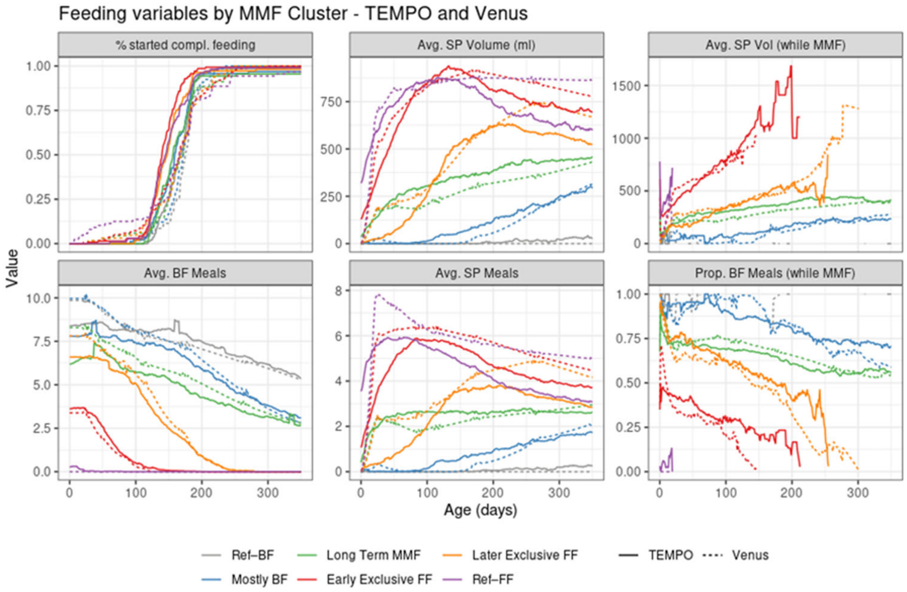

Figure S6. Summary of feeding variables in TEMPO and Venus, per Cluster, from birth (leftmost) to 350 days of age (right most). First row: % started compl. feeding - percentage of subjects that introduced complementary feeding; Avg. SP Volume (ml) - average of estimated daily study product volume intake; Avg. SP Vol (while MMF) - average of estimated study product volume intake in ml per day, only for subjects that are mixed feeding Second row: Avg. BF Meals - average of estimated daily number of daily BF meals; Avg. SP Meals - average of estimated daily number of daily study product meals. Prop. BF Meals (while MMF) - proportion of BF meals from total of BF meals and study product meals.

3. Supplementary material – Tables

Table S1. Median age at introducing complementary feeding (95% confidence limits)

MMF Cluster	TEMPO	Venus
Ref–BF	167 [163, 174]	175 [168, 181]
Mostly BF	159 [154, 168]	172 [166, 181]
Long Term MMF	157 [152, 166]	173 [160, 180]
Later Exclusive FF	148 [139, 153]	168 [159, 180]
Early Exclusive FF	140 [135, 146]	166 [162, 174]
Ref–FF	146 [138, 153]	167 [158, 181]

Table S2. TEMPO – Selected baseline variables by Feeding Group.

	Total (N=855)	Ref–BF (N=141)	Ref–FF (N=102)	MMF (>21 days MMF) (N=432)	Short transition (<21 days MMF) (N=92)	Follow-up < 300 days (N=88)
**Birth weight (grams)**						
Mean (SD)	3323.0 (378.1)	3405.1 (356.0)	3349.7 (451.5)	3296.7 (365.7)	3306.5 (348.7)	3306.7 (396.4)
Range	2229.0 - 4480.0	2620.0 - 4310.0	2500.0 - 4480.0	2229.0 - 4375.0	2355.0 - 4160.0	2415.0 - 4230.0
**Sex**						
Female	422 (49.4%)	72 (51.1%)	53 (52.0%)	207 (47.9%)	45 (48.9%)	45 (51.1%)
Male	433 (50.6%)	69 (48.9%)	49 (48.0%)	225 (52.1%)	47 (51.1%)	43 (48.9%)
**Mother's highest level of education completed**						
N-Miss	1	0	0	1	0	0
High school / trade school or equivalent	344 (40.3%)	42 (29.8%)	63 (61.8%)	155 (36.0%)	42 (45.7%)	42 (47.7%)
Primary school	41 (4.8%)	2 (1.4%)	5 (4.9%)	16 (3.7%)	8 (8.7%)	10 (11.4%)
University degree or higher	469 (54.9%)	97 (68.8%)	34 (33.3%)	260 (60.3%)	42 (45.7%)	36 (40.9%)
**Mother's body mass index (kg/m2), pre-pregnancy**						
N-Miss	7	0	0	4	1	2
Mean (SD)	24.8 (4.7)	24.1 (3.7)	26.4 (5.6)	24.6 (4.6)	24.7 (4.8)	25.0 (4.6)
Range	12.1 - 65.4	18.4 - 40.0	16.7 - 41.5	12.1 - 65.4	15.9 - 43.9	16.8 - 36.8
**Mother's weight (kg), pre-pregnancy**						
N-Miss	6	0	0	3	1	2
Mean (SD)	68.2 (13.9)	66.6 (10.3)	72.8 (17.1)	67.5 (13.8)	68.5 (14.8)	68.4 (13.2)
Range	38.0 - 178.0	47.0 - 100.0	47.0 - 120.0	38.0 - 178.0	46.0 - 130.0	43.0 - 100.0
**Mother's age (years)**						

N-Miss	1	0	0	1	0	0
Mean (SD)	31.7 (4.9)	32.3 (4.6)	31.5 (5.7)	31.9 (4.6)	30.7 (5.1)	30.8 (5.3)
Range	17.0 - 45.0	21.0 - 44.0	17.0 - 45.0	18.0 - 43.0	19.0 - 42.0	19.0 - 44.0
**Race**						
N-Miss	1	0	0	1	0	0
Caucasian	717 (84.0%)	129 (91.5%)	91 (89.2%)	339 (78.7%)	88 (95.7%)	70 (79.5%)
Chinese	103 (12.1%)	8 (5.7%)	5 (4.9%)	77 (17.9%)	1 (1.1%)	12 (13.6%)
Other Asian	15 (1.8%)	2 (1.4%)	2 (2.0%)	6 (1.4%)	0 (0.0%)	5 (5.7%)
Other/Combination	19 (2.2%)	2 (1.4%)	4 (3.9%)	9 (2.1%)	3 (3.3%)	1 (1.1%)
**Mode of delivery**						
Caesarean section	269 (31.5%)	40 (28.4%)	53 (52.0%)	132 (30.6%)	22 (23.9%)	22 (25.0%)
Vaginal (incl. instrumental)	586 (68.5%)	101 (71.6%)	49 (48.0%)	300 (69.4%)	70 (76.1%)	66 (75.0%)
**Number of biological siblings (incl. half brothers/sisters)**						
Mean (SD)	0.9 (1.1)	1.1 (1.0)	0.9 (1.1)	0.9 (1.0)	0.9 (1.0)	1.0 (1.3)
Range	0.0 - 8.0	0.0 - 7.0	0.0 - 7.0	0.0 - 6.0	0.0 - 5.0	0.0 - 8.0
**Mother's any allergy**						
No	302 (35.3%)	49 (34.8%)	40 (39.2%)	154 (35.6%)	28 (30.4%)	31 (35.2%)
Yes	553 (64.7%)	92 (65.2%)	62 (60.8%)	278 (64.4%)	64 (69.6%)	57 (64.8%)
**Both parents any allergy**						
No	694 (81.2%)	114 (80.9%)	86 (84.3%)	340 (78.7%)	75 (81.5%)	79 (89.8%)
Yes	161 (18.8%)	27 (19.1%)	16 (15.7%)	92 (21.3%)	17 (18.5%)	9 (10.2%)

Table S3. TEMPO – Selected baseline variables by MMF Cluster

	Ref–BF (N=141)	Mostly BF (N=94)	Long Term MMF (N=89)	Later Exclusive FF (N=109)	Early Exclusive FF (N=140)	Ref–FF (N=102)
**Birth weight (grams)**						
Mean (SD)	3405.1 (356.0)	3322.9 (361.2)	3306.8 (345.1)	3309.4 (383.4)	3262.9 (368.9)	3349.7 (451.5)
Range	2620.0 - 4310.0	2590.0 - 4110.0	2500.0 - 4310.0	2500.0 - 4375.0	2229.0 - 4220.0	2500.0 - 4480.0
**Sex**						
Female	72 (51.1%)	46 (48.9%)	33 (37.1%)	57 (52.3%)	71 (50.7%)	53 (52.0%)
Male	69 (48.9%)	48 (51.1%)	56 (62.9%)	52 (47.7%)	69 (49.3%)	49 (48.0%)
**Mother's highest level of education completed**						
N-Miss	0	0	1	0	0	0
High school / trade school or equivalent	42 (29.8%)	28 (29.8%)	22 (25.0%)	42 (38.5%)	63 (45.0%)	63 (61.8%)
Primary school	2 (1.4%)	2 (2.1%)	2 (2.3%)	4 (3.7%)	8 (5.7%)	5 (4.9%)
University degree or higher	97 (68.8%)	64 (68.1%)	64 (72.7%)	63 (57.8%)	69 (49.3%)	34 (33.3%)
**Mother's body mass index (kg/m2), pre-pregnancy**						
N-Miss	0	1	2	1	0	0
Mean (SD)	24.1 (3.7)	24.2 (3.9)	24.3 (5.6)	24.0 (3.9)	25.5 (4.7)	26.4 (5.6)
Range	18.4 - 40.0	15.9 - 37.1	16.9 - 65.4	12.1 - 39.4	17.4 - 46.5	16.7 - 41.5
**Mother's weight (kg), pre-pregnancy**						
N-Miss	0	0	2	1	0	0
Mean (SD)	66.6 (10.3)	67.3 (12.6)	66.6 (16.9)	66.0 (12.4)	69.5 (13.3)	72.8 (17.1)
Range	47.0 - 100.0	45.0 - 107.0	45.0 - 178.0	38.0 - 102.0	49.0 - 120.0	47.0 - 120.0
**Mother's age (years)**						
N-Miss	0	0	1	0	0	0
Mean (SD)	32.3 (4.6)	32.1 (4.4)	33.3 (4.1)	31.3 (4.8)	31.4 (4.8)	31.5 (5.7)
Range	21.0 - 44.0	21.0 - 41.0	24.0 - 42.0	19.0 - 43.0	18.0 - 40.0	17.0 - 45.0
**Race**						

N-Miss	0	1	0	0	0	0
Caucasian	129 (91.5%)	78 (83.9%)	56 (62.9%)	93 (85.3%)	112 (80.0%)	91 (89.2%)
Chinese	8 (5.7%)	12 (12.9%)	28 (31.5%)	13 (11.9%)	24 (17.1%)	5 (4.9%)
Other Asian	2 (1.4%)	1 (1.1%)	1 (1.1%)	1 (0.9%)	3 (2.1%)	2 (2.0%)
Other/Combination	2 (1.4%)	2 (2.2%)	4 (4.5%)	2 (1.8%)	1 (0.7%)	4 (3.9%)
**Mode of delivery**						
Caesarean section	40 (28.4%)	22 (23.4%)	29 (32.6%)	33 (30.3%)	48 (34.3%)	53 (52.0%)
Vaginal (incl. instrumental)	101 (71.6%)	72 (76.6%)	60 (67.4%)	76 (69.7%)	92 (65.7%)	49 (48.0%)
**Number of biological siblings (incl. half brothers/sisters)**						
Mean (SD)	1.1 (1.0)	0.9 (0.9)	0.9 (1.2)	0.8 (0.9)	0.9 (1.0)	0.9 (1.1)
Range	0.0 - 7.0	0.0 - 3.0	0.0 - 6.0	0.0 - 4.0	0.0 - 5.0	0.0 - 7.0
**Mother's any allergy**						
No	49 (34.8%)	31 (33.0%)	34 (38.2%)	34 (31.2%)	55 (39.3%)	40 (39.2%)
Yes	92 (65.2%)	63 (67.0%)	55 (61.8%)	75 (68.8%)	85 (60.7%)	62 (60.8%)
**Both parents any allergy**						
No	114 (80.9%)	68 (72.3%)	70 (78.7%)	82 (75.2%)	120 (85.7%)	86 (84.3%)
Yes	27 (19.1%)	26 (27.7%)	19 (21.3%)	27 (24.8%)	20 (14.3%)	16 (15.7%)

Table S4. Venus – Selected baseline variables by Feeding Group

	Total (N=504)	Ref–BF (N=35)	Ref–FF (N=88)	MMF (>21 days MMF) (N=335)	Short transition (<21 days MMF) (N=30)	Follow-up < 300 days (N=16)
**Birth weight (grams)**						
Mean (SD)	3152.1 (349.1)	3209.7 (356.1)	3102.9 (354.0)	3168.8 (351.3)	3051.1 (322.9)	3136.1 (273.0)
Range	2120.0 - 4162.0	2545.0 - 3846.0	2435.0 - 4162.0	2120.0 - 4122.0	2464.0 - 3830.0	2596.0 - 3734.0
**Sex**						
Female	244 (48.4%)	20 (57.1%)	36 (40.9%)	167 (49.9%)	11 (36.7%)	10 (62.5%)
Male	260 (51.6%)	15 (42.9%)	52 (59.1%)	168 (50.1%)	19 (63.3%)	6 (37.5%)
**Mother's highest level of education completed**						
N-Miss	2	0	0	1	0	1
High school / trade school or equivalent	307 (61.2%)	6 (17.1%)	73 (83.0%)	198 (59.3%)	22 (73.3%)	8 (53.3%)
Primary school	16 (3.2%)	1 (2.9%)	8 (9.1%)	5 (1.5%)	1 (3.3%)	1 (6.7%)
University degree or higher	179 (35.7%)	28 (80.0%)	7 (8.0%)	131 (39.2%)	7 (23.3%)	6 (40.0%)
**Mother's body mass index (kg/m2), pre-pregnancy**						
N-Miss	2	0	0	1	0	1
Mean (SD)	22.8 (5.0)	21.0 (2.5)	24.5 (6.8)	22.7 (4.6)	22.6 (4.4)	20.5 (2.7)
Range	15.1 - 47.9	16.9 - 28.2	15.4 - 47.9	15.1 - 44.1	17.4 - 33.3	15.2 - 27.0
**Mother's weight (kg), pre-pregnancy**						
N-Miss	2	0	0	1	0	1
Mean (SD)	57.9 (13.5)	54.2 (8.3)	61.4 (19.0)	57.8 (12.2)	56.4 (12.7)	51.8 (7.5)
Range	35.0 - 145.0	42.0 - 78.0	35.0 - 145.0	35.0 - 120.0	39.0 - 90.0	40.0 - 70.0
**Mother's age (years)**						
Mean (SD)	30.3 (5.0)	29.7 (3.6)	29.2 (6.1)	31.0 (4.6)	27.8 (5.1)	29.1 (5.1)

Range	17.0 - 44.0	19.0 - 38.0	18.0 - 43.0	17.0 - 44.0	19.0 - 36.0	21.0 - 36.0
**Race**						
Chinese	320 (63.5%)	32 (91.4%)	40 (45.5%)	221 (66.0%)	16 (53.3%)	11 (68.8%)
Other Asian	168 (33.3%)	2 (5.7%)	42 (47.7%)	108 (32.2%)	12 (40.0%)	4 (25.0%)
Other/Combination	16 (3.2%)	1 (2.9%)	6 (6.8%)	6 (1.8%)	2 (6.7%)	1 (6.2%)
**Mode of delivery**						
Caesarean section	124 (24.6%)	6 (17.1%)	21 (23.9%)	86 (25.7%)	10 (33.3%)	1 (6.2%)
Vaginal (incl. instrumental)	380 (75.4%)	29 (82.9%)	67 (76.1%)	249 (74.3%)	20 (66.7%)	15 (93.8%)
**Number of biological siblings (incl. half brothers/sisters)**						
N-Miss	1	0	0	1	0	0
Mean (SD)	1.2 (1.2)	0.6 (0.9)	1.5 (1.2)	1.2 (1.2)	0.9 (1.0)	0.9 (1.0)
Range	0.0 - 6.0	0.0 - 3.0	0.0 - 6.0	0.0 - 6.0	0.0 - 4.0	0.0 - 3.0
**Mother's any allergy**						
No	343 (68.1%)	17 (48.6%)	64 (72.7%)	236 (70.4%)	17 (56.7%)	9 (56.2%)
Yes	161 (31.9%)	18 (51.4%)	24 (27.3%)	99 (29.6%)	13 (43.3%)	7 (43.8%)
**Both parents any allergy**						
No	443 (87.9%)	32 (91.4%)	78 (88.6%)	296 (88.4%)	25 (83.3%)	12 (75.0%)
Yes	61 (12.1%)	3 (8.6%)	10 (11.4%)	39 (11.6%)	5 (16.7%)	4 (25.0%)

Table S5. Venus – Selected baseline variables by MMF Cluster

	Ref - BF (N=35)	Mostly BF (N=66)	Long Term MMF (N=68)	Later Exclusive FF (N=64)	Early Exclusive FF (N=137)	Ref - FF (N=88)
**Birth weight (grams)**						
Mean (SD)	3209.7 (356.1)	3184.9 (310.0)	3196.6 (367.1)	3212.3 (297.8)	3127.0 (382.7)	3102.9 (354.0)
Range	2545.0 - 3846.0	2410.0 - 3900.0	2440.0 - 4055.0	2470.0 - 3762.0	2120.0 - 4122.0	2435.0 - 4162.0
**Sex**						
Female	20 (57.1%)	36 (54.5%)	36 (52.9%)	24 (37.5%)	71 (51.8%)	36 (40.9%)
Male	15 (42.9%)	30 (45.5%)	32 (47.1%)	40 (62.5%)	66 (48.2%)	52 (59.1%)
**Mother's highest level of education completed**						
N-Miss	0	0	1	0	0	0
High school / trade school or equivalent	6 (17.1%)	22 (33.3%)	45 (67.2%)	37 (57.8%)	94 (68.6%)	73 (83.0%)
Primary school	1 (2.9%)	0 (0.0%)	0 (0.0%)	0 (0.0%)	5 (3.6%)	8 (9.1%)
University degree or higher	28 (80.0%)	44 (66.7%)	22 (32.8%)	27 (42.2%)	38 (27.7%)	7 (8.0%)
**Mother's body mass index (kg/m2), pre-pregnancy**						
N-Miss	0	0	1	0	0	0
Mean (SD)	21.0 (2.5)	21.7 (3.2)	23.4 (4.6)	22.1 (4.9)	23.2 (5.0)	24.5 (6.8)
Range	16.9 - 28.2	16.5 - 31.6	15.1 - 35.6	15.8 - 44.1	15.4 - 37.7	15.4 - 47.9
**Mother's weight (kg), pre-pregnancy**						
N-Miss	0	0	1	0	0	0
Mean (SD)	54.2 (8.3)	55.4 (9.1)	59.1 (11.6)	56.8 (12.7)	58.9 (13.4)	61.4 (19.0)
Range	42.0 - 78.0	41.0 - 80.0	41.0 - 97.0	38.0 - 120.0	35.0 - 95.0	35.0 - 145.0
**Mother's age (years)**						
Mean (SD)	29.7 (3.6)	31.5 (3.9)	32.0 (4.8)	30.5 (4.5)	30.5 (4.9)	29.2 (6.1)
Range	19.0 - 38.0	21.0 - 44.0	20.0 - 44.0	17.0 - 39.0	20.0 - 42.0	18.0 - 43.0
**Race**						

Chinese	32 (91.4%)	57 (86.4%)	48 (70.6%)	46 (71.9%)	70 (51.1%)	40 (45.5%)
Other Asian	2 (5.7%)	8 (12.1%)	17 (25.0%)	18 (28.1%)	65 (47.4%)	42 (47.7%)
Other/Combination	1 (2.9%)	1 (1.5%)	3 (4.4%)	0 (0.0%)	2 (1.5%)	6 (6.8%)
**Mode of delivery**						
Caesarean section	6 (17.1%)	14 (21.2%)	17 (25.0%)	17 (26.6%)	38 (27.7%)	21 (23.9%)
Vaginal (incl. instrumental)	29 (82.9%)	52 (78.8%)	51 (75.0%)	47 (73.4%)	99 (72.3%)	67 (76.1%)
**Number of biological siblings (incl. half brothers/sisters)**						
N-Miss	0	0	1	0	0	0
Mean (SD)	0.6 (0.9)	0.9 (0.9)	1.4 (1.3)	0.8 (0.8)	1.3 (1.5)	1.5 (1.2)
Range	0.0 - 3.0	0.0 - 3.0	0.0 - 6.0	0.0 - 3.0	0.0 - 6.0	0.0 - 6.0
**Mother's any allergy**						
No	17 (48.6%)	43 (65.2%)	44 (64.7%)	49 (76.6%)	100 (73.0%)	64 (72.7%)
Yes	18 (51.4%)	23 (34.8%)	24 (35.3%)	15 (23.4%)	37 (27.0%)	24 (27.3%)
**Both parents any allergy**						
No	32 (91.4%)	58 (87.9%)	57 (83.8%)	59 (92.2%)	122 (89.1%)	78 (88.6%)
Yes	3 (8.6%)	8 (12.1%)	11 (16.2%)	5 (7.8%)	15 (10.9%)	10 (11.4%)
